# 615. A Year with COVID19 – Experience from the Front Line in a Large Infectious Disease (ID) Clinical Practice

**DOI:** 10.1093/ofid/ofab466.813

**Published:** 2021-12-04

**Authors:** Ronald G Nahass, Angelo Giordano, Edward J McManus

**Affiliations:** 1 ID Care, Hillsborough, New Jersey; 2 IDCare, Randolph, New Jersey

## Abstract

**Background:**

ID Care (IDC) is a large, 43 physician, 74 provider, practice that treats patients in 16 acute care hospitals (ACH) and 120 skilled nursing facilities (SNF) in NJ. March 4, 2021 was the first day a patient with COVID19 seen by IDC. Over the subsequent year IDC evaluated, treated, and tested over 23,000 persons for COVID19. Patients were seen in 2 distinct times - wave 1 (W1) March 5-August 31 and wave 2 (W2) September 1 to March 4. We compare the experience of these 2 waves and report on the year of COVID19 at IDC.

**Methods:**

The administrative data base for IDC was queried for demographic, visit and testing information. A survey of providers was performed to capture incidence of COVID19 and vaccination rates. Daily census logs were used to create epi curves. Comparisons between waves were performed using student T Test or X^2^.

**Results:**

Table 1 provides the comparisons between waves. More patients were seen in W2, however, the number of visits per patient was less, consistent with a shorter length of stay. Fewer patients were seen in SNF in W2 compared to W1. The age and sex distribution between the waves were the same. A total of 8741 molecular tests were performed. Test positivity peaked the week of December 31 at 6.99% and dropped to 0% by May 1 consistent with vaccination and the NJ epidemic curve. During the year of COVID19, 6/74 (8%) clinicians were infected with SARSCoV2. All recovered. Infections in providers were not clearly work-related exposures. 73/74 clinicians were vaccinated.

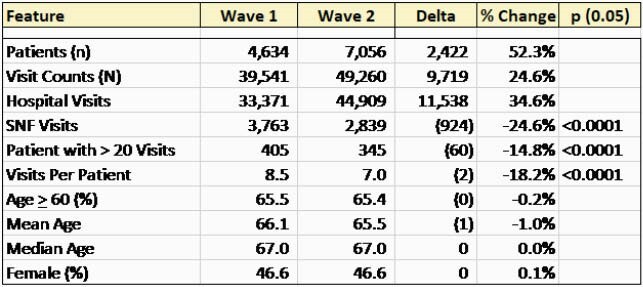

Table 1. Demographics For the Year in COVID19 at ID Care

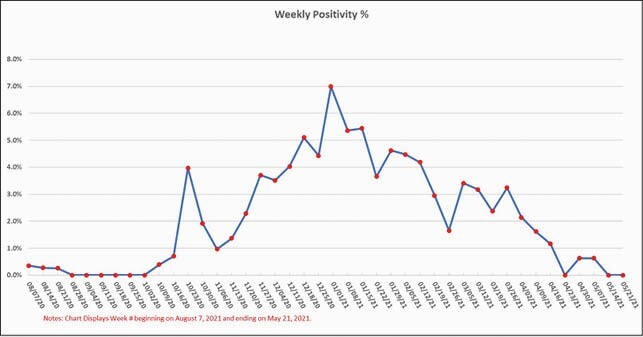

Figure 1. Test Positivity Rate for ID Care

**Conclusion:**

The year of COVID19 occurred in 2 distinct waves. W1 was short and intense. The age and gender distributions were the same between the waves. Even though wave 2 was numerically greater, the cases in SNF were statistically less than the first wave likely from improved IP practice initiated in W1. The numbers of visits per patient, a surrogate for LOS, was statistically less in W2. The decline in test positivity paralleled deployment of vaccination. Despite an intensity of exposure of 158 patients/provider or 1198 visits/provider to SARSCoV2 infected persons only 8% of the clinician staff were infected. ID clinical practice can use electronic databases to help describe regional outbreaks of transmissible disease giving additional perspective across the care continuum. A more usable standard tool would enhance this capacity.

**Disclosures:**

**Ronald G. Nahass, MD**, **Abbvie** (Grant/Research Support, Speaker's Bureau)**Alkermes** (Grant/Research Support)**Gilead** (Grant/Research Support, Speaker's Bureau)**Merck** (Grant/Research Support, Speaker's Bureau)

